# Retraction: Synergistic Antitumor Effects of Novel HDAC Inhibitors and Paclitaxel In Vitro and In Vivo

**DOI:** 10.1371/journal.pone.0344841

**Published:** 2026-03-16

**Authors:** The PLOS One Editors

After this article [1] was published, concerns were raised regarding results presented in Figs 2, 3, 5 and 6. Specifically,

- In the cleaved CPP32 panel in Fig 3C, the upper and lower bands in lanes 5-8 appear similar to each other.
- In the Cleaved CPP32 panel in Fig 5F, the bands in lanes 2 and 3 appear similar to each other.
- Lanes 1-4 in the Fig 6A cytosol mitochondrial marker panel appear similar to lanes 1-4 in the Fig 6A mitochondria β-tubulin panel.
- The following western blot panels appear similar to each other:
  - Fig 3F β tubulin and Fig 5D actin
  - Fig 3A IGROV-1 mitochondrial extract mit. marker and Fig 3A IGROV-1/Pt1 CPP32
  - Fig 3E actin and Fig 5E actin
  - Fig 3C β tubulin and Fig 5G actin
  - Fig 3A IGROV-1/Pt1 actin and Fig 5C actin
- There appear to be vertical discontinuities in several panels in Figs 2, 3, 5 and 6.

The authors did not respond to the editorial request for comment on these concerns.

In light of the above concerns, which call into question the integrity and reliability of [1], the *PLOS One* Editors retract this article.

All authors either did not respond directly or could not be reached.
